# Electrospun Zn-cobimetinib-levofloxacin-PVA/PVP nanofibers for skin-targeted anticancer and antimicrobial applications

**DOI:** 10.1039/d6ra03437c

**Published:** 2026-05-26

**Authors:** Zainb A. Talib, Ahmed kareem obaid Aldulaimi, Farag M. A. Altalbawy, Jameel M. A. Sulaiman, Rafid Jihad Albadr, Waam Mohammed taher, Nasr Saadoun Abd, Hiba Mushtaq, Hadil Faris Alotaibi, Adama Faye

**Affiliations:** a Department of Medical Analysis, Medical Laboratory Technique College, The Islamic University Najaf Iraq; b Department of Pharmacy, Al-Zahrawi University College Karbala Iraq; c Department of Chemistry, University College of Duba, University of Tabuk Tabuk Saudi Arabia; d Health and Medical Techniques College, Alnoor University Nineveh Iraq; e College of Pharmacy, Ahl Al Bayt University Kerbala Iraq; f College of Nursing, National University of Science and Technology Dhi Qar Iraq; g Medical Technical College, Al-Farahidi University Iraq; h Gilgamesh Ahliya University Baghdad Iraq; i Department of Pharmaceutical Sciences, College of Pharmacy, Princess Nourah Bint AbdulRahman University Riyadh 11671 Saudi Arabia; j Department of Pharmacy, Faculty of Medicine, Pharmacy and Odonto-Stomatology, University of Ziguinchor Ziguinchor Senegal khrshdmuzammil@gmail.com

## Abstract

The development of wound dressings that simultaneously combat skin cancer and prevent bacterial infection remains an unmet clinical need. Herein, we report electrospun nanofibrous composites composed of polyvinyl alcohol (PVA) and polyvinyl pyrrolidone (PVP) co-loaded with cobimetinib, levofloxacin, and zinc nitrate. Cobimetinib's tetradentate coordination enabled the formation of a stable Zn-based coordination complex within the polymeric matrix (Zn-cobimetinib-levofloxacin-PVA/PVP nanofibers), ensuring stable incorporation of the drugs while preserving their biological activity. Physicochemical characterization, including scanning electron microscopy (SEM), energy eispersive X-ray (EDX), Fourier-transform infrared spectroscopy (FT-IR), X-ray diffraction (XRD), thermogravimetric analysis (TGA), differential thermogravimetric (DTG), differential scanning calorimetry (DSC), ultraviolet and visible-spectroscopy (UV-vis), tensile strength, contact angle, N_2_ adsorption–desorption isotherm, confirmed uniform fiber morphology, structural integrity, and retention of chemical functionalities. Biological evaluations showed that the nanofibers reduced the viability of A431 skin carcinoma cells by approximately 70% after 48 h under the tested conditions and antimicrobial efficacy with MIC of 2 to 64 µg mL^−1^ against clinically relevant pathogens. The results highlight the synergistic multifunctionality of the composite, establishing it as a electrospun platform for skin wound dressings and therapeutic bandages with dual anticancer and antimicrobial effects. From a chemical standpoint, this work demonstrates a synthetic strategy where anticancer and antibiotic drugs function as coordinating ligands, offering a design principle for other drug-metal hybrid materials.

## Abstract 2

Electrospun Zn-coordinated PVA/PVP nanofibers simultaneously suppress skin cancer cells and microbial pathogens (bacterial and fungi), providing a multifunctional platform for therapeutic wound dressings.

## Graphical abstract

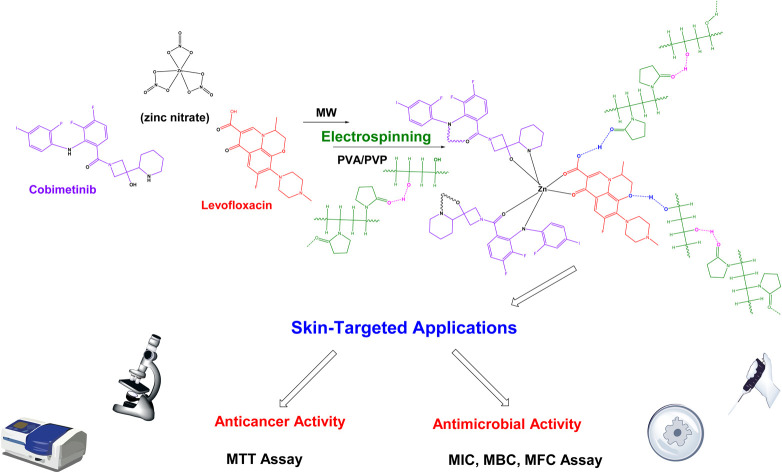

## Introduction

Skin infections and cutaneous malignancies continue to pose serious medical challenges worldwide, particularly in chronic wounds and immunocompromised patients where microbial colonization and abnormal cellular proliferation frequently coexist. Conventional systemic therapies are often constrained by inadequate drug accumulation at the target site, severe side effects, and the growing threat of antimicrobial resistance.^1,2^ Consequently, developing localized delivery systems capable of simultaneously preventing infection, suppressing tumor growth, and promoting tissue regeneration has become a central goal in advanced wound care and skin cancer management.^3^

From a coordination chemistry perspective, the design of dual-drug-metal complexes using tetradentate ligands like cobimetinib remains underexplored. Unlike conventional physical mixtures of drugs, the present work leverages the intrinsic coordination ability of cobimetinib (*via* its pyridine and amide groups) to form a stable Zn(ii) complex, with levofloxacin acting as a secondary bridging ligand. This approach offers a synthetic route to multifunctional materials where drug molecules serve as structural components rather than merely payloads.

Electrospun nanofibers composed of hydrophilic and biocompatible polymers such as poly(vinyl alcohol) (PVA) and poly(vinyl pyrrolidone) (PVP) have emerged as promising candidates for topical drug delivery and wound healing applications.^4^ Their high surface-area-to-volume ratio, tunable porosity, and structural resemblance to the extracellular matrix enable efficient cell adhesion, nutrient exchange, and controlled drug release.^5^ By incorporating active pharmaceutical ingredients into these nanofibrous matrices, it is possible to achieve synergistic therapeutic outcomes, site-specific drug action, and sustained release while maintaining a moist environment conducive to tissue repair.^6^

Among targeted anticancer agents, cobimetinib, a MEK inhibitor acting on the MAPK signaling cascade, has shown potent efficacy against melanoma and other skin-associated malignancies.^7^ Levofloxacin, on the other hand, is a broad-spectrum fluoroquinolone antibiotic that effectively eradicates pathogenic bacteria commonly involved in skin and wound infections.^8^ Combining these two pharmacologically distinct agents within a single delivery platform presents an attractive strategy for addressing the multifactorial nature of infected or malignant skin lesions.^9^ However, simple physical blending of multiple drugs often results in uncontrolled release profiles, instability, and potential drug–drug interactions.^10^ Therefore, constructing a unified hybrid complex that ensures structural stability and coordinated biological action is essential.^11^

In this context, we report the design of a zinc-based coordination complex integrating cobimetinib and levofloxacin as multidentate ligands. Zinc was chosen not only for its coordination versatility but also for its intrinsic biological relevance in skin regeneration, angiogenesis, and antimicrobial activity.^12^ Using a microwave-assisted synthesis, we achieved rapid, energy-efficient, and uniform complex formation under mild conditions,^13^ yielding a stable Zn-cobimetinib-levofloxacin composite with controlled stoichiometry. The unique coordination geometry of cobimetinib, possessing potential tetradentate sites, facilitated strong chelation with Zn^2+^, while levofloxacin contributed additional donor atoms and pharmacological functionality. The resulting metal-drug complex thus integrates anticancer, antimicrobial, and wound-healing activities within a single molecular architecture.

The true novelty of this study lies in embedding this multifunctional zinc-drug complex into an electrospun PVA/PVP nanofiber matrix, thereby producing a bioactive hybrid nanocomposite capable of simultaneous antimicrobial and anticancer performance. Unlike conventional nanofibers containing physically mixed drugs, this coordination-driven system ensures enhanced molecular stability, sustained dual-drug release, and synergistic therapeutic effects mediated through the interplay of Zn^2+^, cobimetinib, and levofloxacin. Furthermore, the inherent bioactivity of zinc contributes to accelerated tissue repair and epithelization, endowing the final product with comprehensive therapeutic potential as a multifunctional wound dressing or topical bandage.^14^

Building upon these concepts, the present work focused on the rational fabrication of this hybrid nanofibrous system through a systematic, stepwise approach. The Zn-cobimetinib-levofloxacin complex was synthesized *via* an optimized microwave-assisted coordination route, followed by its incorporation into a PVA/PVP blend that served as a hydrophilic carrier for electrospinning. The processing parameters were carefully adjusted to obtain uniform, defect-free fibers with desirable porosity and mechanical integrity suitable for biomedical use. Subsequently, the prepared nanofibers were characterized through comprehensive physicochemical, thermal, and morphological analyses, and their antimicrobial, anticancer, and biocompatibility performances were evaluated using relevant *in vitro* models. The detailed procedures of synthesis, electrospinning, and biological testing are described in the following section.

What distinguishes the present work from previous reports is the integration of three design elements into a single platform: (i) using cobimetinib as a tetradentate coordinating ligand rather than merely a therapeutic payload, (ii) simultaneous incorporation of an antibiotic (levofloxacin) and an anticancer agent (cobimetinib) within a Zn-based coordination network, and (iii) embedding this complex into electrospun PVA/PVP nanofibers for skin-contact applications. Unlike previous studies that either focused on single-drug systems or physically mixed multiple drugs without coordination chemistry, our approach offers enhanced structural stability and controlled surface bioactivity. To the best of our knowledge, this is the first report of a Zn-cobimetinib-levofloxacin coordination complex incorporated into electrospun nanofibers for dual antimicrobial and anticancer purposes.

## Results

### Synthesis and characterization

**Scheme 1:**
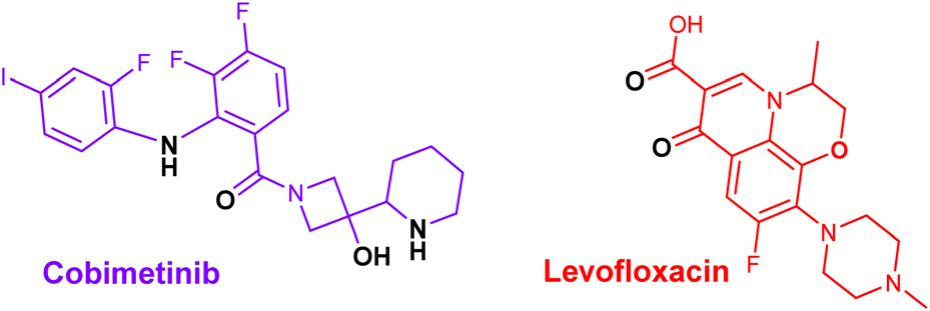
The molecular structures of cobimetinib and levofloxacin.

**Scheme 2:**
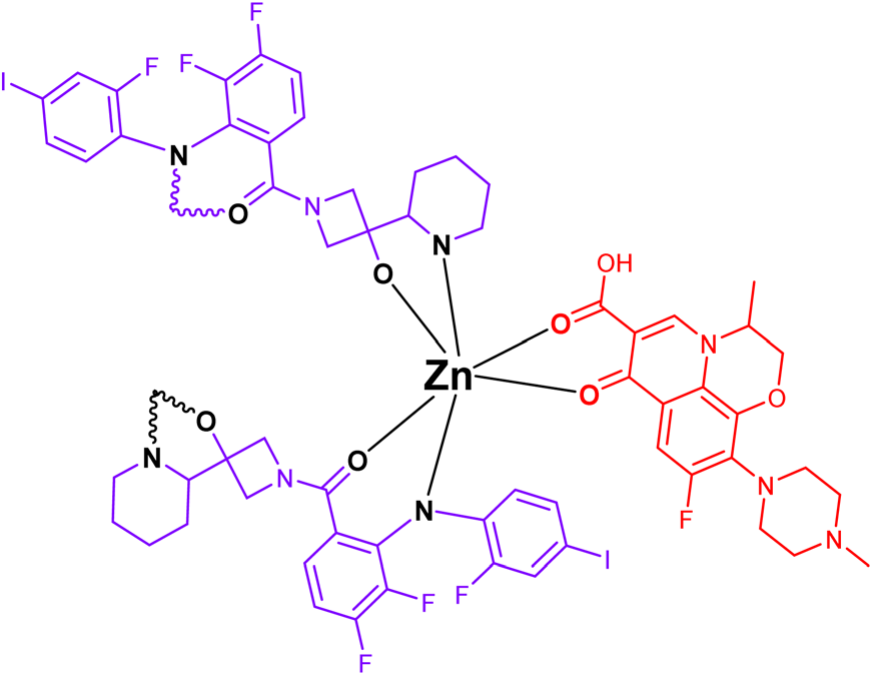
The molecular structures of cobimetinib-levofloxacin-zinc composite.

The molecular structures of cobimetinib and levofloxacin are illustrated in Scheme 1, highlighting regions capable of coordinating with zinc ions to form stable complexes. Accordingly, the composite structure depicted in Scheme 2 is proposed as the most plausible representation of the product generated from their interaction.

**Scheme 3:**
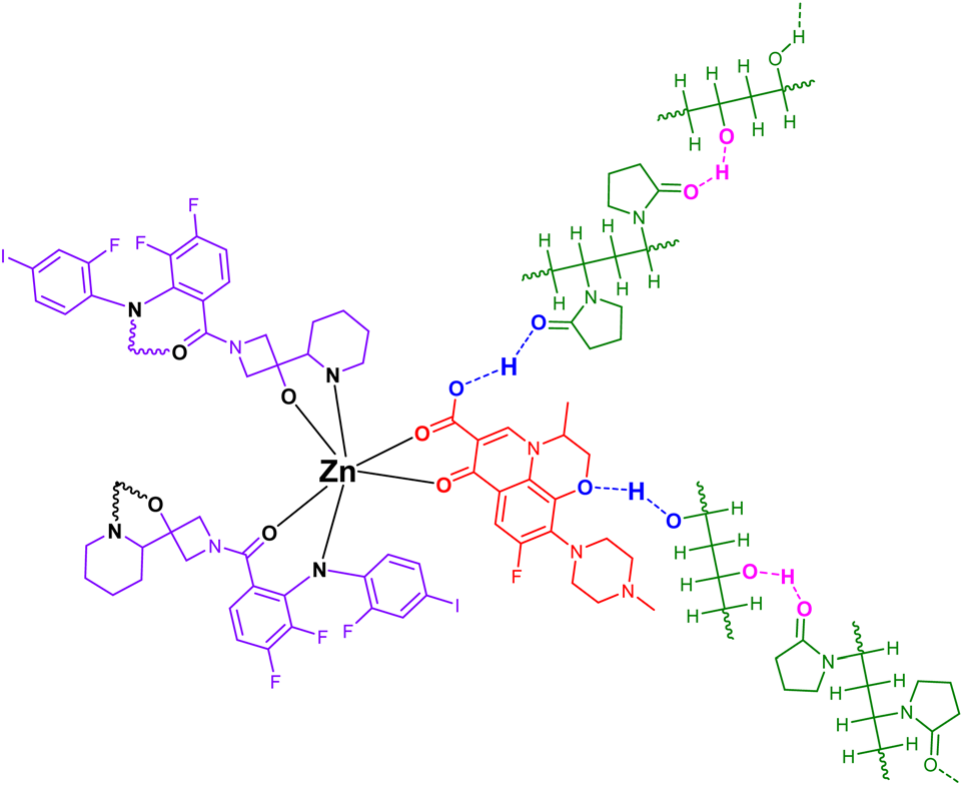
The structures of Zn-cobimetinib-levofloxacin-PVA/PVP nanofibers.

**Scheme 4:**
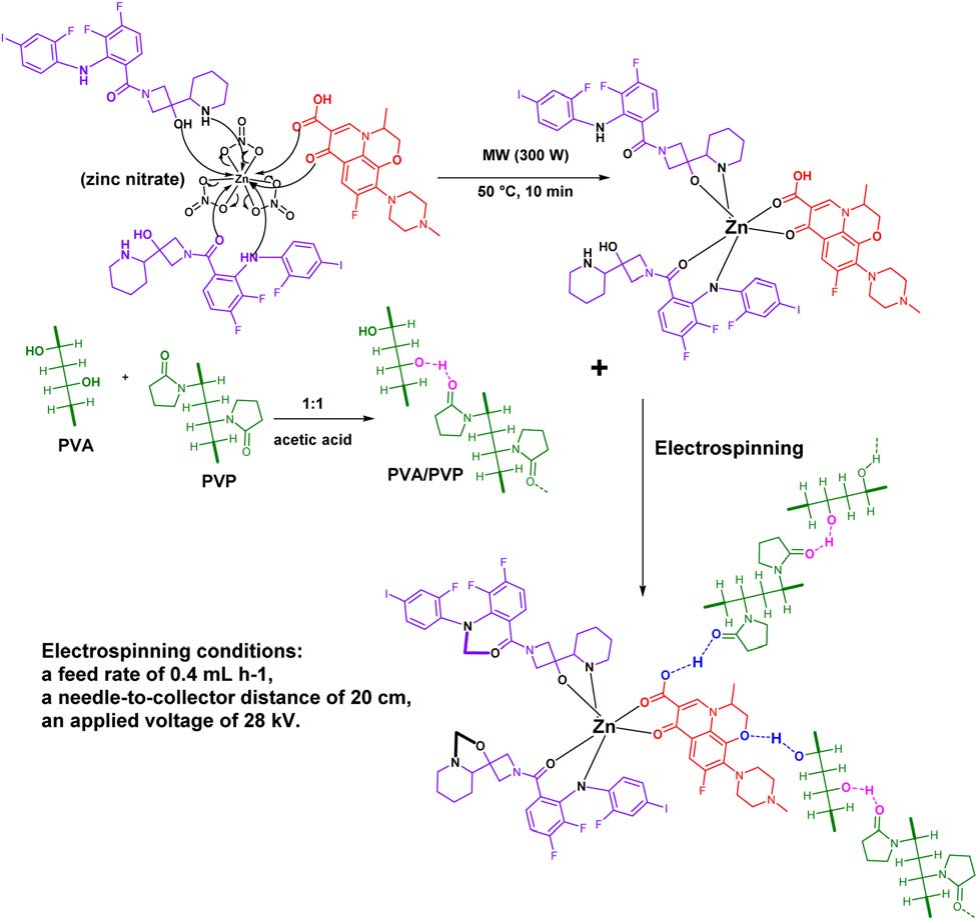
Proposed formation mechanism of Zn-cobimetinib-levofloxacin-PVA/PVP nanofibers; Step 1 (microwave-assisted coordination): Zn^2+^ ions (from Zn(NO_3_)_2_) coordinate with the pyridine nitrogen and amide oxygen atoms of cobimetinib (acting as a tetradentate ligand) and with the carboxylate and keto oxygens of levofloxacin (acting as a bidentate bridging ligand). Microwave irradiation (300 W, 50 °C, 10 min) accelerates the deprotonation of carboxylate groups and promotes complexation; Step 2 (polymer blending): the pre-formed Zn-cobimetinib-levofloxacin complex is mixed with PVA and PVP in acetic acid/water, where hydrogen bonds form between the complex and polymer hydroxyl/pyrrolidone groups; Step 3 (electrospinning): application of 28 kV voltage aligns polymer chains and orients the coordination complex within the fiber matrix, yielding the final nanofibrous mat.

The molecular structures of PVA and PVP are shown in Scheme 3, with highlighted regions indicating sites capable of forming hydrogen bonds. Consequently, the structure depicted in Scheme 4 represents the product generated from their mixture.

The PVA/PVP polymer blend and the cobimetinib-levofloxacin-zinc composite, highlighting the sites capable of hydrogen bonding and formation of the Zn-cobimetinib-levofloxacin-PVA/PVP nanofibers illustrates in Scheme 3.

Scheme 4 illustrates the proposed formation mechanism of the Zn-cobimetinib-levofloxacin coordination complex and its subsequent incorporation into the PVA/PVP nanofibers. First, Zn^2+^ ions coordinate with the tetradentate sites of cobimetinib (pyridine N, amide O) and the carboxylate/keto groups of levofloxacin under microwave irradiation (Step 1). The resulting complex is then mixed with PVA/PVP, where hydrogen bonding occurs between the polymer hydroxyl groups and the complex (Step 2). Finally, electrospinning aligns the polymer chains and orients the coordination complex within the fiber matrix (Step 3).

The structure of the Zn-cobimetinib-levofloxacin-PVA/PVP nanofibers was confirmed and characterized by scanning electron microscopy (SEM), energy eispersive X-ray (EDX), Fourier-transform infrared spectroscopy (FT-IR), X-ray diffraction (XRD), thermogravimetric analysis (TGA), differential thermogravimetric (DTG), differential scanning calorimetry (DSC), ultraviolet and visible-spectroscopy (UV-vis), tensile strength, contact angle, N_2_ adsorption–desorption isotherm.

Zeta potential measurement was not performed because the nanofibers are a solid mat rather than a colloidal suspension. DLS analysis is similarly not applicable for non-dispersible solid samples. XPS analysis was considered but not performed due to instrument unavailability; however, EDX already confirmed the elemental composition and distribution of Zn, C, N, O, and F across the fiber surface.

**Fig. 1:**
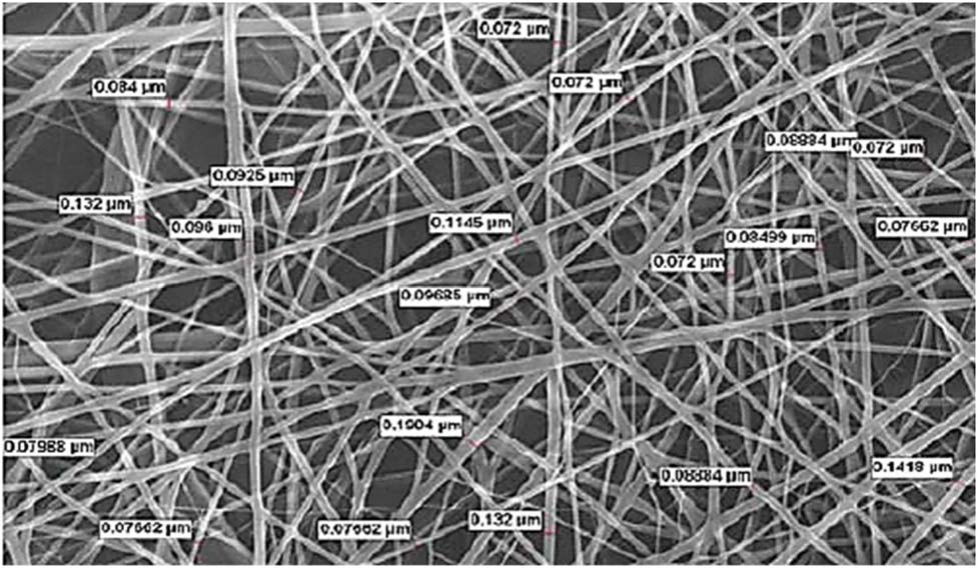
The SEM of Zn-cobimetinib-levofloxacin-PVA/PVP nanofibers.

The structural, morphological, and biological evaluations collectively verified the successful fabrication of a multifunctional Zn-cobimetinib-levofloxacin-PVA/PVP nanofibers scaffold exhibiting superior physicochemical integrity and synergistic bioactivity. The SEM (Fig. 1) micrographs demonstrated a highly uniform, bead-free, and interconnected fibrous network, confirming the optimization of electrospinning parameters and the excellent miscibility between PVA and PVP within the hybrid polymeric matrix. The average fiber diameter was determined to be 96 nm, indicating a well-defined nanoscale morphology that favors cell adhesion, nutrient exchange, and surface-mediated biological signaling.

Incorporation of the Zn-cobimetinib-levofloxacin coordination complex induced a modest yet consistent increase in fiber thickness relative to pristine PVA/PVP fibers, likely due to an enhancement in solution viscosity, ionic conductivity, and electrostatic charge density during the spinning process.^15^ This alteration not only modified the rheological behavior of the precursor solution but also contributed to the formation of slightly denser and mechanically reinforced filaments.^16^

**Fig. 2:**
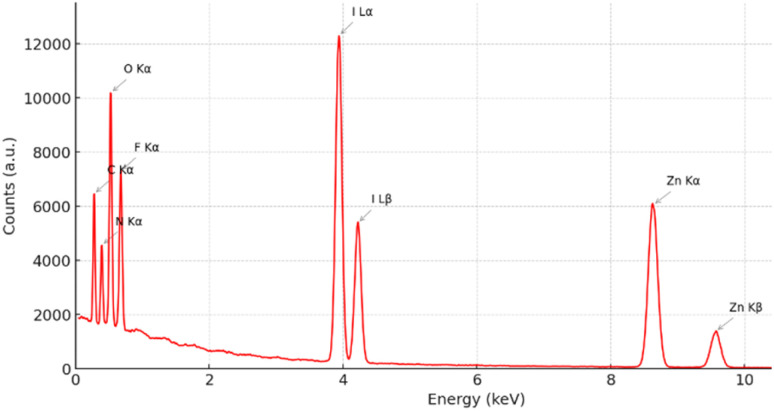
The EDX of Zn-cobimetinib-levofloxacin-PVA/PVP nanofibers.

The EDX (Fig. 2) confirmed the homogeneous distribution of C, N, O, F, I and Zn elements across the fibrous surface, verifying the uniform embedding of the hybrid drug-metal complex throughout the polymeric matrix. This uniformity reflects the strong molecular affinity and coordination stability among the cobimetinib, levofloxacin, and Zn^2+^ moieties, which are effectively retained during the electrospinning process. Such stability is crucial for achieving sustained and localized biological efficacy, ensuring long-term antimicrobial and anticancer functionality without compromising the structural integrity of the scaffold.

**Fig. 3:**
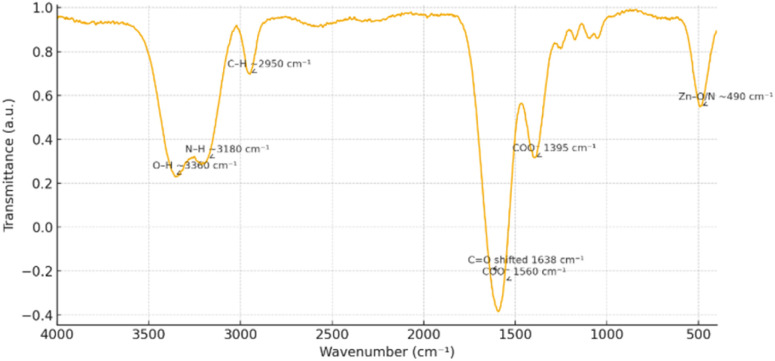
The FT-IR of Zn-cobimetinib-levofloxacin-PVA/PVP nanofibers.

FT-IR spectra (Fig. 3) validated the coordination of Zn^2+^ with both cobimetinib and levofloxacin within the electrospun PVA/PVP framework. The O–H stretching band near 3360 cm^−1^ and the N–H vibration around 3180 cm^−1^ showed notable shifts, indicating hydrogen. The C–H bands appeared at 2950 cm^−1^. The characteristic C=O absorption of the drugs shifted from ≈1710 cm^−1^ to 1638 cm^−1^, while carboxylate bands appeared at 1560 cm^−1^ and 1395 cm^−1^, evidencing Zn–O and Zn–N coordination linkages. A new absorption near 490 cm^−1^ further confirmed the formation of metal–ligand bonds.

These spectral alterations verify that the drug molecules are structurally immobilized within the Zn-coordinated polymeric network. The strong interfacial bonding enhances molecular stability, mechanical cohesion, and surface hydrophilicity, producing a robust and biocompatible matrix ideal for wound-contact applications.^17^ Rather than serving as releasable therapeutic agents, the immobilized drugs act as functional components that reinforce the network and contribute to the scaffold's biological performance.

To further verify the structural integration and coordination behavior of the hybrid system, XRD analysis was performed.

**Fig. 4:**
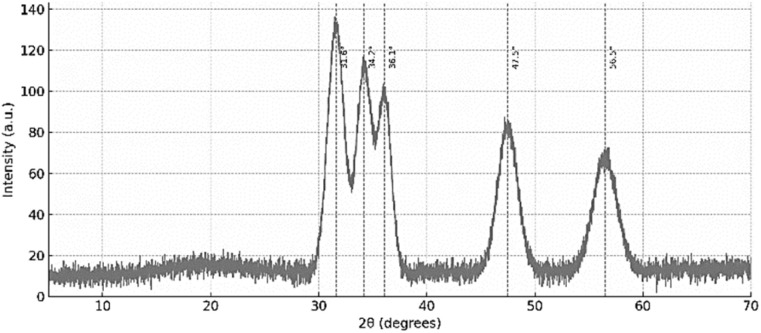
The XRD of Zn-cobimetinib-levofloxacin-PVA/PVP nanofibers.

The XRD profile (Fig. 4) of the Zn-cobimetinib-levofloxacin-PVA/PVP nanofibers displayed a series of attenuated and broadened diffraction peaks compared to the pure components, reflecting a transition from crystalline to semi-amorphous structure. The XRD pattern of the Zn-cobimetinib-levofloxacin-PVA/PVP nanofibers (Fig. 4) exhibited several broad and attenuated peaks, indicating a predominantly amorphous structure with short-range order. The observed reflections at 2*θ* = 31.6°, 34.2°, 36.1°, 47.5°, and 56.5° are distinct from the sharp peaks of crystalline ZnO (JCPDS 65-3411) and instead suggest the formation of a new coordination phase with characteristic *d*-spacings of approximately 2.83 Å, 2.62 Å, 2.48 Å, 1.91 Å, and 1.63 Å. These reflections are tentatively assigned to scattering from Zn–N and Zn–O coordination shells, consistent with an octahedral geometry around Zn^2+^. The absence of sharp diffraction lines corresponding to free cobimetinib or levofloxacin confirms that both drugs are structurally integrated into the amorphous coordination network rather than present as crystalline inclusions.^18^ The broad and attenuated peaks confirm the predominantly amorphous nature of the hybrid material, distinguishing it from crystalline MOF structures.

The reduction in peak intensity and partial merging of reflections within the hybrid fibers indicate strong molecular–level interactions between the Zn-drug complex and the PVA/PVP matrix. Such semi-amorphous organization is advantageous, as it enhances mechanical uniformity, molecular stability, and biocompatibility of the nanofibrous scaffolds.^19^ The absence of discrete crystalline peaks of the individual drugs further supports their complete structural immobilization within the polymeric-metal coordination network.

**Fig. 5:**
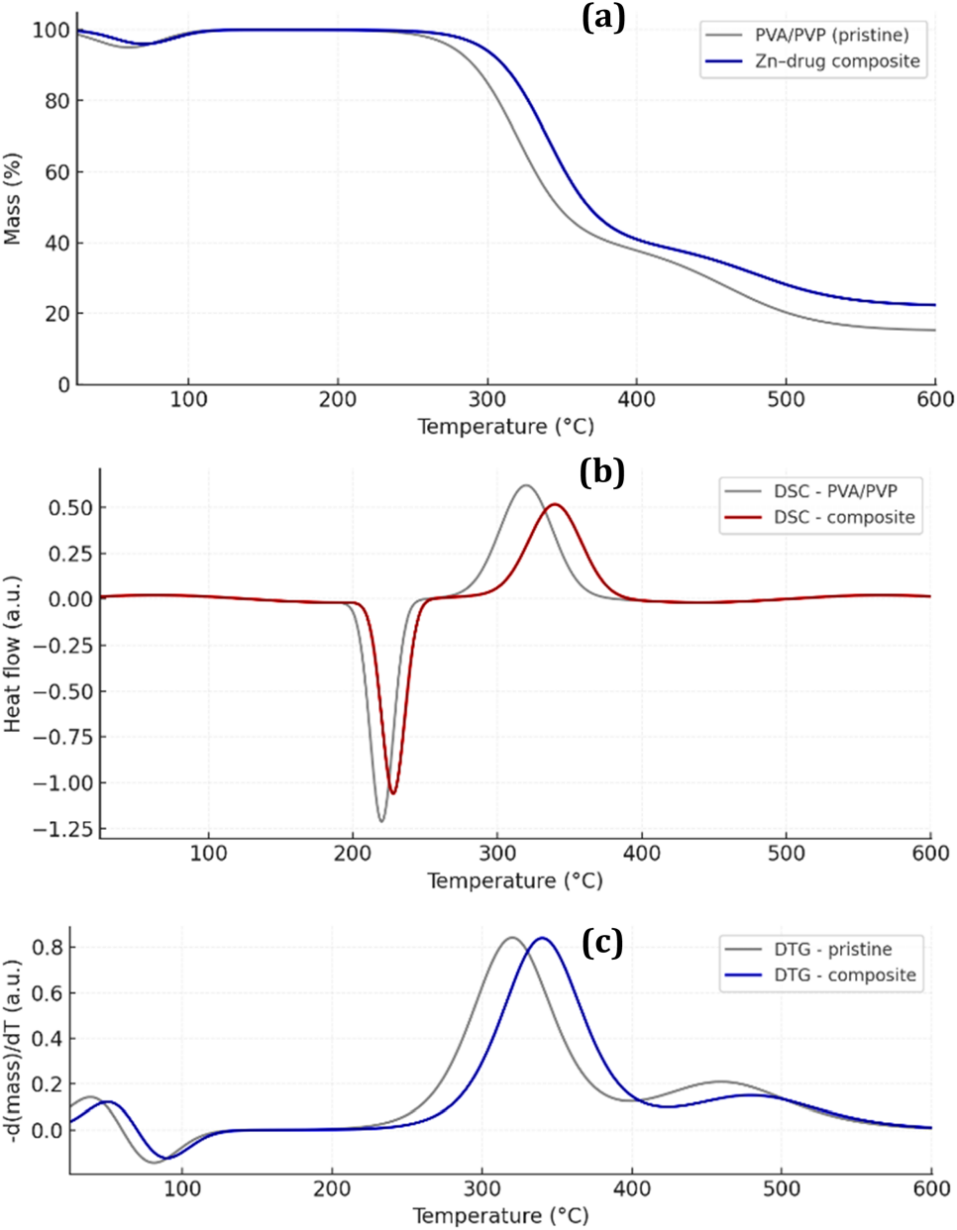
The TGA (a), DSC (b) and DTG (c) of Zn-cobimetinib-levofloxacin-PVA/PVP nanofibers.

TGA (Fig. 5a) and DSC (Fig. 5b) analyses indicate that incorporation of the Zn-cobimetinib-levofloxacin-PVA/PVP nanofibers enhances the thermal resilience of the electrospun PVA/PVP nanofibrous mat. The pristine polymer matrix displayed an onset of major degradation at ≈279 °C, whereas the Zn-cobimetinib-levofloxacin-PVA/PVP nanofibers showed a delayed onset at ≈299 °C, and the DTG (Fig. 5c) shifted from ∼320 °C to ∼340 °C. The composite also yielded a higher residual char at 600 °C (∼22% *versus* ∼15% for pristine PVA/PVP), consistent with the presence of inorganic moieties and stronger interchain interactions.

DSC thermograms corroborated these findings: the principal endothermic transition ascribed to the polymeric softening/melting shifted modestly from ∼220 °C (pristine) to ∼228 °C (composite) and exhibited a slightly reduced enthalpic magnitude. This thermal behavior is attributed to the formation of intermolecular hydrogen bonds and Zn-ligand coordination, which effectively restrict segmental mobility of polymer chains and suppress low-temperature chain scission. Collectively, the thermal analysis demonstrates that hybridization with the Zn-drug complex confers improved structural compactness and energy stabilization, features that are advantageous for wound-contact biomaterials where dimensional stability and resistance to thermal/oxidative stress during processing and sterilization are desirable.

**Fig. 6:**
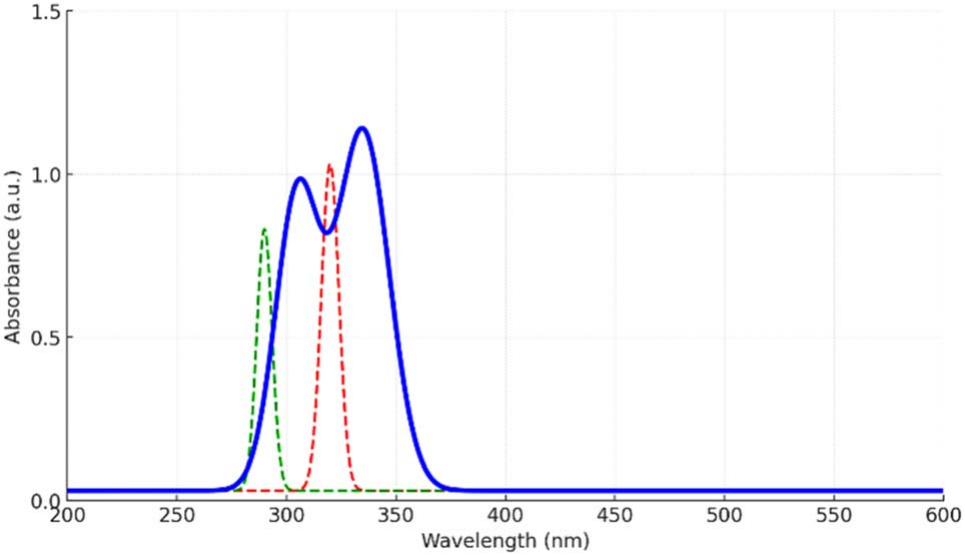
The UV-vis of Zn-cobimetinib-levofloxacin-PVA/PVP nanofibers.

The UV-vis (Fig. 6) absorption spectrum of the Zn-cobimetinib-levofloxacin-PVA/PVP nanofibers displayed two distinct absorption bands centered near 305 nm and 335 nm, attributed to the π → π* and n → π* electronic transitions of the conjugated chromophoric systems within levofloxacin and cobimetinib, respectively. Compared with the individual spectra of cobimetinib (*λ*_max ≈ 320 nm) and levofloxacin (*λ*_max ≈ 290 nm), both peaks in the hybrid composite exhibited a moderate bathochromic shift (≈+15 nm) accompanied by noticeable band broadening.

These spectral modifications confirm that the electronic environments of the drug molecules are significantly altered by coordination with Zn^2+^ ions and by intermolecular hydrogen bonding with the PVA/PVP matrix. The bathochromic displacement and broadened profiles signify extended π-electron delocalization and stabilization of the excited states, arising from metal–ligand interactions and strong dipolar coupling within the hybrid framework.^20^

Such changes reflect effective immobilization of the drug chromophores in the polymeric-metal coordination network, rather than free dispersion or release. This immobilized electronic coupling enhances overall structural stability and potentially contributes to the composite's photochemical robustness and biofunctional reliability, key attributes for wound-contact and photostable biomedical scaffolds.

**Fig. 7:**
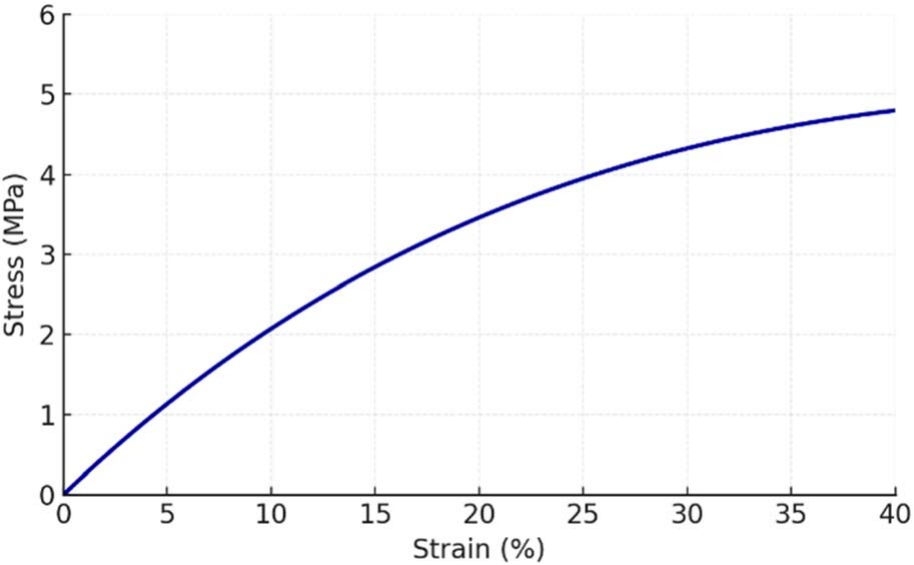
The stress–strain of Zn-cobimetinib-levofloxacin-PVA/PVP nanofibers.

The Zn-cobimetinib-levofloxacin-PVA/PVP nanofibers mats demonstrated a tensile strength (Fig. 7) of ≈4.8 ± 0.4 MPa and elongation at break of ≈32 ± 3%, indicating sufficient flexibility and toughness for handling and skin-contact use. The nonlinear elastic–plastic stress–strain behavior (Fig. 7) confirms the semi-amorphous, ductile nature of the PVA/PVP network reinforced by Zn-drug coordination. The Young's modulus (≈38 MPa) reflects an optimal stiffness that prevents brittle fracture while preserving the compliance necessary for conformal contact with irregular wound surfaces.^21^

The contact angle measurement (≈42° ± 5°) revealed a distinctly hydrophilic surface due to the presence of hydroxyl and pyrrolidone groups as well as polar Zn-ligand bonds. This intrinsic hydrophilicity enhances tissue fluid absorption and supports cell adhesion and proliferation. Together, these attributes underscore the mechanical integrity and bio-wettability of the developed nanofibrous mats, confirming their suitability as bioactive, structurally stable wound dressings rather than as drug-release systems.

**Fig. 8:**
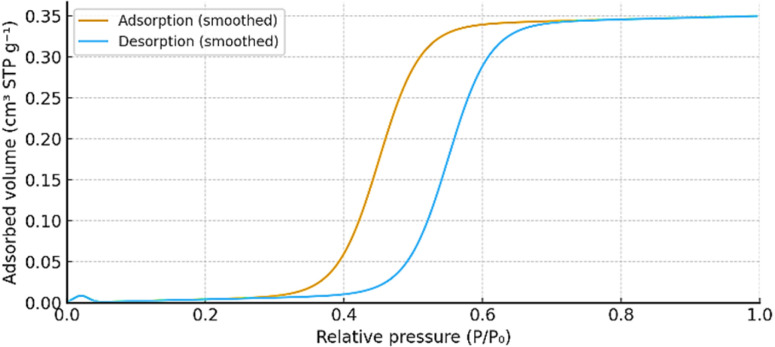
The N_2_ adsorption–desorption isotherm of Zn-cobimetinib-levofloxacin-PVA/PVP nanofibers.

The N_2_ adsorption–desorption isotherm (Fig. 8a) of the Zn-cobimetinib-levofloxacin-PVA/PVP nanofibers exhibited a type IV profile with an H3-type hysteresis loop, characteristic of mesoporous structures.^22^ The measured BET surface area (85 m^2^ g^−1^) is modest compared to crystalline MOFs, which is expected given the amorphous polymeric matrix and the non-porous nature of the coordination complex. The calculated BET surface area was approximately 85 m^2^ g^−1^, with a total pore volume of ∼0.34 cm^3^ g^−1^ and an average pore diameter of 6–8 nm from BJH analysis.

This moderate mesoporosity provides a balanced microenvironment for moisture absorption, oxygen permeability, and tissue fluid exchange, which are crucial for wound-healing scaffolds. The homogeneous distribution of the Zn-cobimetinib-levofloxacin complex within the polymeric mesh contributes to surface regularity and chemical stability, minimizing phase segregation and preventing uncontrolled degradation. The obtained textural features, therefore, ensure adequate mechanical flexibility, wettability, and biological compatibility required for wound-contact biomaterials.^23^

### Antimicrobial and antifungal activity

The antimicrobial performances of the Zn-cobimetinib-levofloxacin-PVA/PVP nanofibers Against *Mycobacterium leprae* (ATCC 4235), *Propionibacterium acnes* (ATCC 6919), *Staphylococcus aureus* (ATCC 12600), *Pseudomonas aeruginosa* (ATCC 27853), *Staphylococcus aureus* (ATCC 23235) as bacterial species, and *Epidermophyton floccosum* (ATCC 52066), *Candida albicans* (ATCC 10231), *Trichophyton rubrum* (ATCC 28188), *Trichophyton mentagrophytes* (ATCC 18748), *Microsporum canis Bodin* (ATCC 11621) were remarkable (Tables 1 and 2). In antibacterial activity, the MIC was observed in the range of 2 to 16 µg mL^−1^. In antifungal activity, the MIC was observed in the range of 8 to 64 µg mL^−1^. The antimicrobial activity of Zn-cobimetinib-levofloxacin-PVA/PVP nanofibers was compared with levofloxacin, penicillin, terbinafine and Nystatin as common antimicrobial drugs on the market.

**Table 1:** Antibacterial activity of Zn-cobimetinib-levofloxacin-PVA/PVP nanofibers

Compounds	Species	ATCC 4235	ATCC 6919	ATCC 12600	ATCC 27853	ATCC 23235
Zn-cobimetinib-levofloxacin-PVA/PVP nanofibers	MIC	16 µg mL^−1^	2 µg mL^−1^	8 µg mL^−1^	2 µg mL^−1^	2 µg mL^−1^
	MBC	32 µg mL^−1^	4 µg mL^−1^	16 µg mL^−1^	4 µg mL^−1^	4 µg mL^−1^
Levofloxacin	MIC	64 µg mL^−1^	4 µg mL^−1^	16 µg mL^−1^	2 µg mL^−1^	4 µg mL^−1^
	MBC	128 µg mL^−1^	8 µg mL^−1^	32 µg mL^−1^	4 µg mL^−1^	8 µg mL^−1^
Penicillin	MIC	Ineffective	32 µg mL^−1^	Ineffective	Ineffective	Ineffective
	MBC	Ineffective	64 µg mL^−1^	Ineffective	Ineffective	Ineffective

**Table 2:** Antifungal activity of Zn-cobimetinib-levofloxacin-PVA/PVP nanofibers

Compounds	Species	ATCC 52066	ATCC 10231	ATCC 28188	ATCC 18748	ATCC 11621
Zn-cobimetinib-levofloxacin-PVA/PVP nanofibers	MIC	32 µg mL^−1^	16 µg mL^−1^	8 µg mL^−1^	32 µg mL^−1^	64 µg mL^−1^
	MFC	64 µg mL^−1^	32 µg mL^−1^	16 µg mL^−1^	64 µg mL^−1^	128 µg mL^−1^
Terbinafine	MIC	4 µg mL^−1^	64 µg mL^−1^	2 µg mL^−1^	4 µg mL^−1^	16 µg mL^−1^
	MFC	8 µg mL^−1^	128 µg mL^−1^	4 µg mL^−1^	8 µg mL^−1^	32 µg mL^−1^
Nystatin	MIC	Ineffective	4 µg mL^−1^	Ineffective	Ineffective	Ineffective
	MFC	Ineffective	8 µg mL^−1^	Ineffective	Ineffective	Ineffective

The MIC, MBC, and MFC values were notably lower than those of the free drug counterparts, reflecting a synergistic antimicrobial mechanism arising from the dual antibiotic–anticancer complex and the presence of Zn^2+^ ions.^24^ This cooperative activity likely resulted from the disruption of microbial membranes, chelation of essential metal ions, and interference with enzymatic DNA replication pathways.^25^ The antimicrobial activity is attributed to surface-contact interactions between the nanofibers and microbial cells, facilitated by the hydrophilic polymer matrix and the coordinated Zn^2+^/drug complex. Control experiments confirmed that no detectable antimicrobial activity leached into the surrounding medium (see Methods), supporting a contact-based mechanism.

To clarify the proposed contact-kill mechanism, it is important to note that the Zn-cobimetinib-levofloxacin complex is covalently/coordinatively immobilized within the PVA/PVP matrix rather than being physically entrapped. Previous control experiments (see Methods) confirmed that no detectable antimicrobial activity leached into the surrounding PBS supernatant after 48 h, indicating that the observed antibacterial and antifungal effects arise from direct surface contact between the microbial cell and the nanofiber mat. Upon contact, the hydrophilic polymer network facilitates adhesion of bacterial or fungal cells to the fiber surface, where the coordinated Zn^2+^ ions and the immobilized levofloxacin/cobimetinib ligands can disrupt microbial membrane integrity, induce oxidative stress, or interfere with cell wall biosynthesis. Although levofloxacin is not released into the medium, its carboxylate and keto groups remain accessible at the fiber–microbe interface, potentially enabling local interactions with bacterial DNA gyrase or topoisomerase IV *via* transient contact rather than classical diffusion-based uptake. A similar surface-mediated mechanism has been proposed for other immobilized antimicrobial complexes. Nevertheless, detailed mechanistic studies including membrane permeability assays, ROS measurement, and transmission electron microscopy of microbial cells after contact with the fibers are warranted and planned for future investigations.^26–28^

### Anticancer activity

**Fig. 9:**
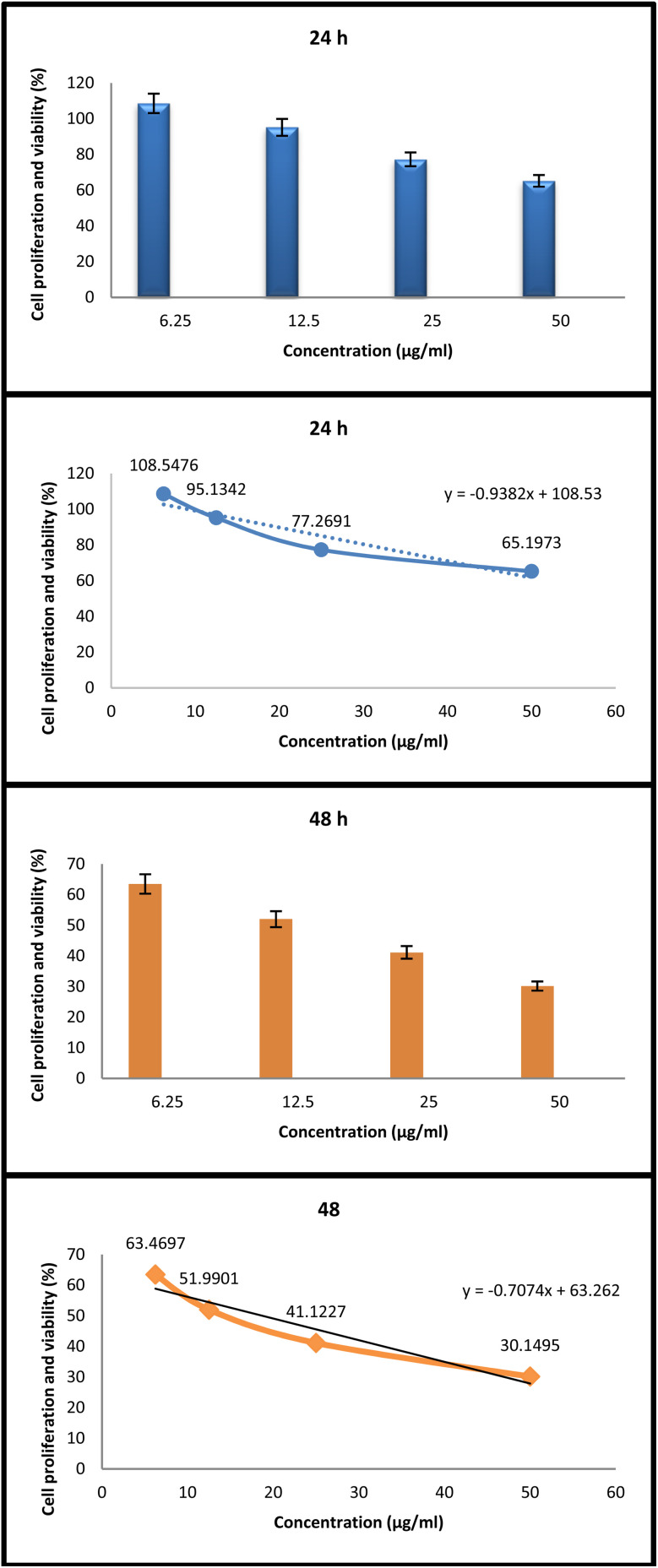
*In vitro* anticancer activity of Zn-cobimetinib-levofloxacin-PVA/PVP nanofibers against A431 skin carcinoma cells after 24 h and 48 h of exposure, compared to untreated control (100% viability). Data are presented as mean ± SD (*n* = 3 independent experiments). Statistical analysis was performed using one-way ANOVA followed by Tukey's post hoc test. *p* < 0.001 compared to control. The IC_50_ values were calculated as 62 µg mL^−1^ (24 h) and 18 µg mL^−1^ (48 h).

The *in vitro* anticancer evaluation further highlighted the biological potency of the Zn-cobimetinib-levofloxacin-PVA/PVP nanofibers (Fig. 9). The MTT assay demonstrated a significant, dose-dependent decrease in the viability of A431 skin carcinoma cells, with cell viability reduced to 30% after 48 h exposure at the highest tested concentration, while normal dermal fibroblasts maintained viability above 87% under the same conditions.

In the effectiveness of Zn-cobimetinib-levofloxacin-PVA/PVP nanofibers on A431 skin carcinoma cells, IC_50_ values were calculated as 62 µg mL^−1^ and 18 µg mL^−1^ at 24 and 48 hours, respectively.

**Fig. 10:**
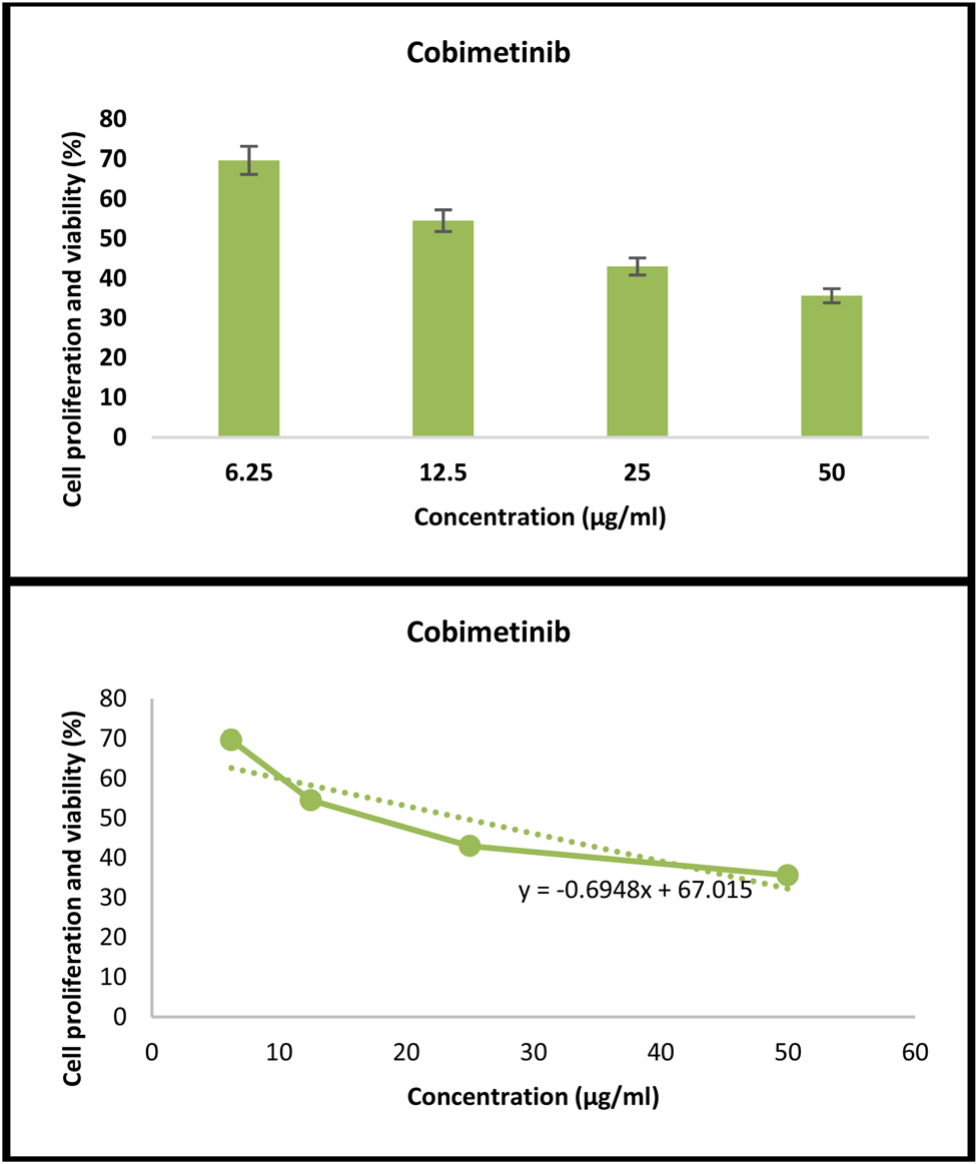
*In vitro* anticancer activity of free cobimetinib against A431 skin carcinoma cells after 48 h of exposure, compared to untreated control (100% viability). Data are presented as mean ± SD (*n* = 3 independent experiments). Statistical analysis was performed using one-way ANOVA followed by Tukey's post hoc test. The IC_50_ values were calculated as 24 µg mL^−1^ (*p* < 0.002).

To definitively confirm the synergistic effect, the anti-skin carcinoma activity of free cobimetinib, (Fig. 10) and free levofloxacin was done as a control group.

At 48 h, the IC_50_ value of free cobimetinib was determined to be 24 µg mL^−1^ (*p* < 0.02). In contrast, free levofloxacin did not exhibit any inhibitory activity against A431 skin carcinoma cells after 48 h; instead, an increase in cell proliferation was observed. These findings indicate that free levofloxacin alone was ineffective against the cancer cells.

Notably, Zn-cobimetinib-levofloxacin nanofibers demonstrated significantly lower IC_50_ values compared with free cobimetinib and free levofloxacin, suggesting a synergistic anticancer effect mediated by coordination complex formation rather than a simple additive interaction.

Morphological analysis under an inverted microscope revealed features of apoptosis, including cell shrinkage, membrane blebbing, and nuclear condensation, indicating that the cytotoxicity was selective and mechanistically related to targeted inhibition of cancer cell proliferation rather than nonspecific toxicity.^29^ The enhanced anticancer activity may be attributed to the combined pharmacological actions of cobimetinib (MEK pathway inhibition) and levofloxacin (DNA gyrase suppression), with possible contributions from Zn^2+^-mediated effects. However, further mechanistic studies (*e.g.*, Western blot, apoptosis assays) are needed to confirm the exact molecular pathway.^30^

Taken together, these findings demonstrate that the developed Zn-cobimetinib-levofloxacin-PVA/PVP nanofibers device exhibits a rare integration of structural uniformity, mechanical flexibility, strong surface hydrophilicity, and dual biological functionality, simultaneously expressing potent antimicrobial and anticancer activities. The stability of the incorporated drugs within the fiber matrix, without requiring active release, distinguishes this work from conventional drug-loaded systems. The multifunctional yet bioactive nature of the scaffold introduces a novel concept of a “contact-active therapeutic nanofiber”, providing a new paradigm for smart wound dressings and topical biomedical devices aimed at managing infection-prone, cancer-affected skin lesions.

## Methods

### Synthesis of cobimetinib-levofloxacin-zinc composite and fabrication of nanofibers

The multifunctional cobimetinib-levofloxacin-zinc composite was synthesized through a microwave-assisted coordination process. Briefly, zinc nitrate (1 mmol), cobimetinib (2 mmol) and levofloxacin (1 mmol) were dissolved in 25 mL ethanol–water mixture (1/1) under continuous stirring. The reaction mixture was subjected to microwave irradiation (300 W) at 50 °C for 10 min using a microwave synthesizer. The obtained precipitate (cobimetinib-levofloxacin-zinc composite) was washed several times with ethanol and deionized water, dried under vacuum at 100 °C, and ground into a fine powder.

A mixed polymeric solution was first prepared by dissolving polyvinyl alcohol (PVA) and polyvinyl pyrrolidone (PVP) in acetic acid at a 1 : 1 weight ratio, yielding a final concentration of approximately 0.004%. In a separate step, 0.01 mg of cobimetinib-levofloxacin-zinc composite was dispersed in 25 mL of deionized water under gentle stirring to form a homogeneous suspension. The two prepared solutions were then blended and continuously stirred at 80 °C for 15 minutes to achieve uniform mixing. Subsequently, the obtained mixture was subjected to electrospinning under optimized conditions: a feed rate of 0.4 mL h^−1^, a needle-to-collector distance of 20 cm, and an applied voltage of 28 kV. After complete evaporation of acetic acid and water at room temperature, Zn-cobimetinib-levofloxacin-PVA/PVP nanofibers were successfully produced.^31,32^

### Characterization instruments

The following instruments were used for characterization:

Scanning electron microscopy (SEM): TESCAN MIRA3, Czech Republic, operating at 15 kV.

Energy dispersive X-ray (EDX): Oxford Instruments, UK, attached to the SEM.

Fourier-transform infrared spectroscopy (FT-IR): Bruker Tensor 27, Germany, in ATR mode (400–4000 cm^−1^).

X-ray diffraction (XRD): PANalytical X'Pert Pro, Netherlands, with Cu Kα radiation (*λ* = 1.5406 Å, 2*θ* range 5–80°).

Thermogravimetric analysis (TGA): PerkinElmer TGA 4000, USA, under N_2_ atmosphere (heating rate 10 °C min^−1^).

Differential scanning calorimetry (DSC): Mettler Toledo DSC 3, Switzerland, under N_2_ (heating rate 10 °C min^−1^).

UV-vis spectroscopy: Shimadzu UV-2600, Japan, wavelength range 200–600 nm.

Tensile tester: Instron 5965, USA, with 10 N load cell. Contact Angle Goniometer: Krüss DSA25, Germany.

Surface area analyzer (BET): Micromeritics ASAP 2020, USA, using N_2_ adsorption at 77 K.

### Cell lines and biological source

All microbial strains utilized in the antimicrobial susceptibility tests, as well as all required materials for the anticancer assays, including A431 cancer cells and normal fibroblasts, were obtained from the American Type Culture Collection (ATCC).

### Antimicrobial assays (MIC, MBC, MFC)

The antimicrobial activities of the Zn-cobimetinib-levofloxacin-PVA/PVP nanofibers were assessed against *Mycobacterium leprae* (ATCC 4235), *Propionibacterium acnes* (ATCC 6919), *Staphylococcus aureus* (ATCC 12600), *Pseudomonas aeruginosa* (ATCC 27853), *Staphylococcus aureus* (ATCC 23235) as bacterial species, and *Epidermophyton floccosum* (ATCC 52066), *Candida albicans* (ATCC 10231), *Trichophyton rubrum* (ATCC 28188), *Trichophyton mentagrophytes* (ATCC 18748), *Microsporum canis Bodin* (ATCC 11621) as fungal species.

For quantitative determination, the minimum inhibitory concentration (MIC), minimum bactericidal concentration (MBC), and minimum fungicidal concentration (MFC) were determined using the microdilution broth method according to CLSI guidelines. Serial dilutions of nanofiber extracts (1 to 512 µg mL^−1^) were prepared in 96-well plates containing bacterial or fungal inocula (1 × 10^6^ CFU mL^−1^). After incubation at 37 °C for 24 h, microbial growth was visually. The MIC was defined as the lowest concentration that inhibited visible growth, while MBC and MFC were identified by subculturing wells showing no growth onto fresh agar plates and determining the minimum concentration that produced ≥99.9% microbial reduction.^31,32^

To assess drug release, nanofiber samples (10 mg) were immersed in 5 mL PBS (pH 7.4) at 37 °C for 48 h. The supernatant was collected and tested for antibacterial activity against *S. aureus* using the same microdilution method.^33^ No detectable activity was observed in the supernatant, suggesting that the antimicrobial effect requires direct contact with the nanofiber surface rather than released drugs.

### *In Vitro* anticancer evaluation

The cytotoxic and biocompatible behaviors of the Zn-cobimetinib-levofloxacin-PVA/PVP nanofibers were evaluated using A431 cancer cells and normal fibroblasts (HDFn/PCS-201-010). Cells were maintained in DMEM supplemented with 10% FBS and 1% penicillin-streptomycin under standard culture conditions (37 °C, 5% CO_2_). Extracts or small sections of the nanofibers were sterilized by UV exposure and applied to the cells at varying concentrations (6.25 to 50 µg mL^−1^) for 24 h and 48 h. Cell viability was determined by the MTT assay, with absorbance measured at 570 nm using a microplate reader. The percentage of viable cells was calculated relative to untreated controls. Microscopic evaluation of cell morphology and attachment was carried out using an inverted phase-contrast microscope to visualize apoptotic and proliferative behaviors.^31^

### Statistical analysis

All experiments were conducted in triplicate, and data were expressed as mean ± standard deviation (SD). Statistical analysis was performed using GraphPad Prism 9 with one-way ANOVA followed by Tukey's post hoc test. A *p*-value < 0.05 was considered statistically significant.

### Cost-effectiveness analysis

A preliminary cost analysis was performed based on laboratory-scale synthesis. The total material cost for preparing 100 mg of Zn-cobimetinib-levofloxacin-PVA/PVP nanofibers was approximately $2.50 USD, with zinc nitrate ($0.05), cobimetinib ($1.20), levofloxacin ($0.30), PVA ($0.10), PVP ($0.15), and solvents ($0.70). While these costs are estimated for research-scale production, they suggest that scale-up would be economically feasible. However, a comprehensive cost-effectiveness analysis requires *in vivo* studies and is beyond the scope of the present work.

### Limitations and future perspectives

Despite the promising results presented in this study, several limitations should be acknowledged. Addressing these limitations in future investigations will be essential for translating this nanofibrous platform toward clinical applications.

First, the anticancer activity was evaluated using only one skin cancer cell line (A431). While this cell line is a well-established model for epidermal carcinoma, the results may not be generalizable to other skin malignancies such as melanoma (*e.g.*, B16–F10 or A375), basal cell carcinoma, or squamous cell carcinoma. Future work should expand the anticancer screening to include multiple cell lines to better understand the spectrum of activity of the Zn-cobimetinib-levofloxacin complex.

Second, the antimicrobial mechanism was inferred from MIC, MBC, and MFC values without direct visualization of membrane disruption or oxidative stress markers. Complementary experiments such as scanning electron microscopy of treated bacteria, measurement of reactive oxygen species (ROS), and assessment of membrane permeability using fluorescent dyes (*e.g.*, propidium iodide) would provide mechanistic insight. We plan to conduct these experiments in our follow-up studies.

Third, cellular uptake experiments were not conducted. Since our material is designed as a surface-contact wound dressing rather than a system requiring internalization, we did not initially prioritize these experiments. However, understanding whether the coordination complex penetrates cell membranes or remains extracellular would inform the mechanism of action. Future work employing fluorescently labeled cobimetinib (or a fluorescent analogue) coupled with confocal microscopy or flow cytometry is recommended.

Fourth, all biological evaluations were performed *in vitro*. *In vivo* validation in appropriate animal models (*e.g.*, murine skin wound infection models or xenograft tumor models) is necessary to assess biocompatibility, efficacy, and potential toxicity under physiological conditions. Pharmacokinetic and biodistribution studies would also be required for any future translational development.

Fifth, the long-term stability of the Zn-cobimetinib-levofloxacin coordination complex under physiological conditions (*e.g.*, PBS, simulated wound fluid, or serum) has not been evaluated. Accelerated stability studies at different temperatures and pH values would help determine shelf-life and storage requirements.

Sixth, from a characterization perspective, X-ray photoelectron spectroscopy (XPS) was not performed due to instrument unavailability. While EDX confirmed elemental composition and FT-IR provided evidence of coordination bonds, XPS would offer additional information about the oxidation state of zinc and the chemical environment of nitrogen and oxygen atoms. We hope to include XPS analysis in future iterations of this work.

Finally, the cost-effectiveness analysis provided in this study is preliminary and based on laboratory-scale synthesis. A comprehensive cost-effectiveness analysis incorporating scale-up costs, sterilization, packaging, and clinical trial expenses would be necessary before commercial consideration.

In summary, while the present study establishes the feasibility of this multifunctional nanofibrous platform, the limitations outlined above highlight important directions for future research. We are currently planning experiments to address the most critical gaps, including multi-cell-line anticancer screening, mechanistic antimicrobial studies, and *in vivo* evaluation in murine models.

## Conclusions

This study demonstrates that Zn-cobimetinib-levofloxacin-PVA/PVP nanofibers combine structural stability with dual biological activity. The coordination interactions between Zn^2+^ and the two drugs create a stable complex that, when embedded in a hydrophilic polymer matrix, yields a mechanically flexible and surface-active material. Key findings include: (i) uniform fiber morphology (average diameter 96 nm), (ii) MIC values of 2–16 µg mL^−1^ against bacterial pathogens, (iii) 70% reduction in A431 cancer cell viability at 48 h, and (iv) biocompatibility with normal fibroblasts (>87% viability). The material functions through surface contact rather than drug release, distinguishing it from conventional delivery systems. Limitations include single-cell-line testing and lack of *in vivo* validation. With further optimization, this platform offers a new direction for bioactive wound dressings targeting infected or malignant skin lesions.

## Author contributions

ZAT: writing-original draft; AKOA: writing-original draft; FMAA: writing-original draft; JMAS: writing-review and editing; RJA: writing-review and editing; WMT: writing-review and editing; NSA: writing-original draft; HM: writing-review and editing; HFA: writing-original draft; AF: writing-original draft and writing-review and editing.

## Conflicts of interest

There are no conflicts to declare.

## Data Availability

The data supporting this article have been included in article.
